# Hemodynamics and tissue oxygenation during balanced anesthesia with a high antinociceptive contribution: an observational study

**DOI:** 10.1186/2047-0525-3-9

**Published:** 2014-10-30

**Authors:** Jaap Jan Vos, Marieke Poterman, Laura N Hannivoort, Victor W Renardel De Lavalette, Michel MRF Struys, Thomas WL Scheeren, Alain F Kalmar

**Affiliations:** 1 Department of Anesthesiology, University of Groningen, University Medical Center Groningen, Groningen, The Netherlands; 2 Department of Ophthalmology, University of Groningen, University Medical Center Groningen, Groningen, The Netherlands; 3 Department of Anaesthesiology and Intensive care medicine, Maria Middelares hospital, Ghent, Belgium

**Keywords:** Propofol, Remifentanil, Cardiac output, Fluid therapy, Balanced anesthesia, Tissue oxygenation, Norepinephrine

## Abstract

**Background:**

In particular surgical conditions, a balanced anesthesia with a high-antinociceptive contribution is required. This may induce cardiovascular impairment and thus compromise tissue oxygenation. In this prospective observational study, we investigated the hemodynamic stability and tissue oxygen saturation (StO_2_) in 40 patients with a high-antinociceptive general anesthesia, goal-directed fluid therapy, and norepinephrine. In addition, optimal surgical conditions and safe and fast emergence are pivotal parts of anesthetic management.

**Methods:**

In high-antinociceptive propofol/remifentanil anesthesia with bispectral index (BIS) between 40 and 60, norepinephrine was administered to maintain mean arterial pressure (MAP) above 80% of individual baseline. Fluid was administered if the ∆ plethysmographic waveform amplitude exceeded 10%. Surgical and recovery conditions, hemodynamic responses, and tissue oxygenation were investigated.

**Results:**

Mean (SD) StO_2_ at the left thenar eminence increased from 83 (6)% before to 86 (4)% 20 min after induction of anesthesia (*p* <0.05). Cardiac index dropped from 3.0 (0.7) to 2.1 (0.4) L min^-1^ (*p* <0.05), MAP from 109 (16) to 83 (14) mm Hg, and heart rate from 73 (12) to 54 (8) bpm (*p* <0.05). Thirteen out of 40 patients received a fluid bolus. The median (range) norepinephrine administration rate was 0.05 (0.0–0.10) μg kg^-1^ min^-1^. After complete akinesia in all patients during surgery, a median (IQR) extubation time of 311 (253–386) s was observed.

**Conclusions:**

This high-antinociceptive balanced anesthesia with goal-directed fluid and vasopressor therapy adequately preserved StO_2_ and hemodynamic homeostasis.

**Trial registration:**

ISRCTN20153044

## Background

Induction to a level of anesthesia adequate to tolerate laryngoscopy requires a combination of hypnotics and analgesics that may induce significant hemodynamic suppression attributed to peripheral vasodilatation, loss of venous tone causing reduction in preload and venous return, and to a lesser extent, a decreased myocardial contractility. After intubation, the sudden decrease in nociceptive stimuli often results in hypotension and frequently requires administration of vasoactive medication if surgical stimulation remains absent for a significant time. These agents may however jeopardize hemodynamic stability and tissue oxygenation [1]. Yet, particular surgical interventions require a sustained high level of analgesia, although only occasional but intense nociceptive stimuli occur in between long periods of low stimulation. In these fluctuating surgical conditions, maintaining a stable anesthesia (which can, among other variables, be reflected by a BIS-value between 40 and 60) may be challenging in view of the fluctuating noxious stimuli. A reliable method to assess the quality of analgesia level is complete tolerance to laryngoscopy, which reflects an equal tolerance to typical surgical stimuli [2].

In addition, many of these surgical interventions have only limited analgesic needs once the procedure is finished making the pharmacological profile of remifentanil suitable for this purpose.

In most surgical conditions, a sound approach to compensate for alternating stimuli would be decreasing the level of analgesia. However, in many procedures where continuous muscle relaxants are undesirable and complete akinesia is nonetheless required, an alternative strategy must be pursued while addressing obvious concerns regarding hemodynamic disturbances and awareness [3].

Furthermore, a safe and fast emergence from anesthesia with avoidance of sudden increases in intra-ocular or middle-ear pressure during extubation [4,5], a full cooperative patient soon after surgery and a concise recovery period is often a pivotal part of the anesthetic management.

Whilst all these desired anesthetic requirements are being addressed, adequate tissue perfusion and oxygenation must be preserved. Only relying on the arterial blood pressure to maintain adequate tissue oxygenation is insufficient, yet most advanced hemodynamic monitoring devices are moderately to highly invasive and are associated with rare but serious complications limiting their value in many patients [6-8]. In the surgical settings described above (with a substantial percentage of day-case surgery), non-invasive monitoring (i.e., providing sufficient information without the risk of inducing complications) is mandatory. The Inspectra® device (Hutchinson Technology, Hutchinson, KS, USA) can be used to continuously monitor the tissue oxygen saturation (StO_2_) non-invasively on the thenar muscle using near-infrared spectroscopy (NIRS) [9] while the Nexfin® monitor (Edwards Lifesciences, Irvine, CA, USA) permits non-invasive measurement of various hemodynamic variables in a beat-to-beat fashion [9-14]. The aim of this observational study was to describe the adequacy of tissue oxygenation and hemodynamic stability together with the quality of the surgical conditions and emergence from anesthesia in a tightly-specified balanced anesthesia with a high-antinociceptive contribution combined with a goal-directed fluid and vasopressor therapy.

## Methods

After the Institutional Ethics Committee approval (Ref: 2012.052, Ethics Committee, University Medical Center Groningen, Groningen, The Netherlands), 40 consecutive adult patients scheduled for a day-case surgery under general anesthesia were recorded and written informed consent was asked for data-analysis. The study was registered at Current Controlled Clinical Trials (ISRCTN20153044).

### Anesthetic management

One gram of Paracetamol was administered orally prior to admission to the OR. Intravenous access was secured using an 18G IV catheter in a left large forearm vein, and a continuous background infusion of crystalloids was started at 90 ml hr^-1^. Anesthesia was induced with remifentanil (1 μg kg^-1^), propofol (1–3 mg kg^-1^), and cisatracurium (0.1 mg kg^-1^).

In patients with preexisting cardiovascular comorbidities such as hypertension, reported peripheral or coronary artery disease in whom post-induction hypotension—defined as a 20% decrease [15,16] in baseline mean arterial pressure (MAP)—can be expected, a norepinephrine (10 μg) single bolus was co-administered prophylactically.

After tracheal intubation, the lungs were ventilated in a volume controlled mode with a tidal volume of 8 ml kg^-1^ lean body weight with a mixture of O_2_/air (F_i_O_2_ 0.4) and 5 cm H_2_O PEEP. The respiratory rate was adjusted to keep an end-tidal CO_2_ pressure between 4.5 and 5.5 kPa.

Anesthesia was maintained with propofol (4 mg kg^-1^ hr^-1^) and remifentanil (0.25 μg kg^-1^ min^-1^) and adequate doses of hypnotics and analgesics to tolerate laryngoscopy [2,17] and to keep BIS between 40 and 60. If necessary, these doses could be increased in 30% steps. In addition, norepinephrine was infused as required to keep MAP above 80% of individual baseline (preoperative ambulatory pressure) [15,16].

The level of respiratory induced variation in the pulse oximetry plethysmographic waveform amplitude (∆POP) [18] was assessed 2 min after intubation; if it was found to be above 10%, hypovolemia was assumed and a fluid bolus of 500 ml colloid solution (Voluven®, Fresenius, Bad Homburg, Germany) was administered.

Administration of propofol, remifentanil, and norepinephrine was stopped simultaneously when the surgical procedure was finished. The trachea was extubated under continuous suction only after the patients were consciously responding and after they deeply inhaled upon verbal command. The extubation time, defined as the time between cessation of the syringe pumps and the moment of extubation, was recorded together with the incidence of any coughing reflex upon extubation. Any patient movement during the procedure was recorded. Two hours after recovery from anesthesia, patients were explicitly asked for any (adverse) experience in the peri-anesthetic period.

### Monitoring

In addition to standard monitoring, a bispectral index (BIS) sensor (Aspect Medical Systems, Norwood, MA, USA) was placed on the forehead [19,20]. The non-invasive Inspectra® device (Model 650, Hutchinson Technology, Hutchinson, KA, USA) measures the peripheral StO_2_ based on near-infrared spectrometry [9]. This device emits near-infrared light (600–800 nm) to the tissue, which then undergoes changes in its spectrum, mostly depending on the degree of microvascular hemoglobin saturation. The probe can be placed on different anatomical locations; but it was placed on the left thenar eminence because of the limited fat-layer with little inter-individual variation [9]. The anesthetist was blinded for StO_2_ readings. The Nexfin® monitor (model 1) is a non-invasive advanced hemodynamic monitoring device with a working principle that is based on the volume clamp method using a photoplethysmograph and an inflatable cuff placed around the intermediate phalanx [21]. This device provides continuous calculation of arterial blood pressure, systemic vascular resistance, stroke volume, cardiac output (CO), and heart rate in a beat-to-beat fashion [10-14].

### Data registration and analysis

The Philips MP70 anesthesia monitor (Philips, Eindhoven, Netherlands) was connected to a PC, and all patient data were recorded at a 1-s interval using Rugloop II data-manager software (Demed, Temse, Belgium). The time and dose of all administered drugs were recorded in the Rugloop system, together with any noteworthy events or patient responses. The following variables were recorded: SpO_2_, StO_2_, heart rate (HR), MAP, CO, Cardiac Index (CI), and BIS. The electronic data were imported into Microsoft Excel 2010® (Microsoft, Redmond, WA, USA). After a graphical representation, a visual inspection of the data plots was performed to correct obvious atypical values caused by artifacts.

### Statistics

All statistics were performed using Microsoft Excel 2010® and PASW Statistics 18 (SPSS Inc, Chicago, IL, USA). Data were expressed as mean (SD), median (range), or number of patients (%). Normality of continuous variables was assessed using the Kolmogorov-Smirnov test. Continuous repetitive data before induction, and at 20 and 60 min thereafter were compared using the paired *t* test. Statistical significance level was set at *p* <0.05.

## Results

A total of 40 consecutive patients were screened for eligibility and subsequently enrolled.

Forty consecutive patients scheduled for ophthalmic surgery under general anesthesia were recorded. Mean (SD) age was 64 (13) years, weight was 82 (14) kg, and height was 176 (10) cm. There were 11, 23, and 6 patients of ASA classifications I, II, and III respectively. The median (range) duration of surgery was 73 (30–184) min. No use of neuromuscular reversal agents was necessary. In two patients (5%), an increase in the dosage of both propofol and remifentanil was required, whereas in one patient (2.5%), only the dosage of propofol was increased.

Overall, the median (range) administration rate of norepinephrine was 0.05 (0.00–0.10) μg kg^-1^ min^-1^. Thirty-two patients were given a prophylactic dose of 10 μg norepinephrine at induction of anesthesia. Twenty-one patients were administered 0.05 μg kg^-1^ min^-1^ norepinephrine continuously, while in eight patients, this administration rate could be either reduced to 0.025 μg kg^-1^ min^-1^ or stopped. In two patients, the administration rate was increased to 0.075 μg kg^-1^ min^-1^ and in one patient to 0.1 μg kg^-1^ min^-1^. Eight patients did not receive norepinephrine at all. Thirteen of the forty patients showed a ∆POP above 10% and were subsequently administered 500 ml fluid.

### Evolution of hemodynamic variables and bispectral index

**Figure 1:**
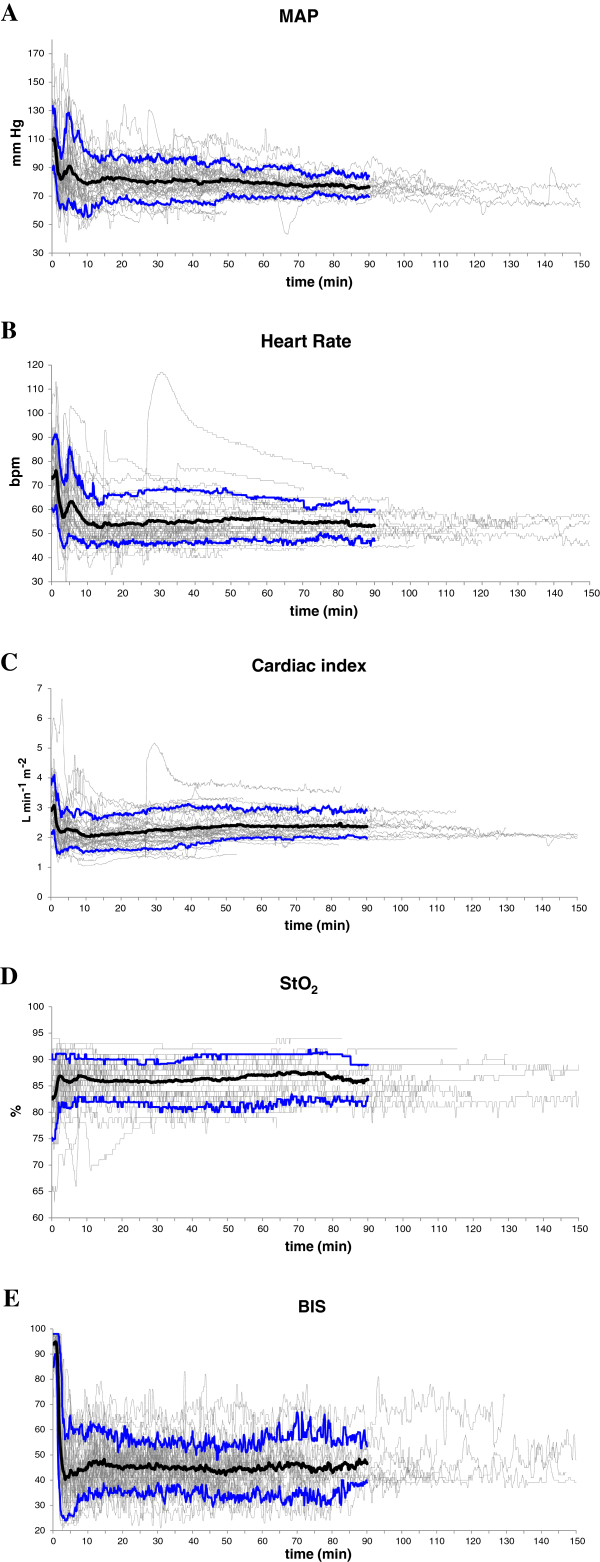
**Evolution of individual values (grey), mean value (black), and 10th and 90th percentile (blue). (A)** Mean arterial pressure. **(B)** Heart rate. **(C)** Cardiac index. **(D)** Tissue oxygen saturation. **(E)** Bispectral index. Values are shown from the moment of induction of anesthesia up to the end of the procedure. Mean and percentiles are shown for the first 90 minutes.

**Figure 2:**
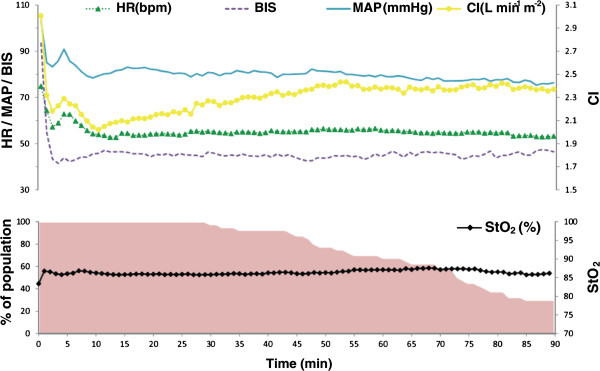
**Time course of HR (bpm), BIS, MAP (mmHg), CI (L min**^**-1**^ **m**^**-2**^**), and StO**_**2 **_**(%).** The variables are shown as mean values from induction of anesthesia up to 90 minutes thereafter. Furthermore, the percentage of the population at each time point that is still included is shown.

Figure 1A,B,C,D,E shows the time course of both the individual and mean values (in bold) of the investigated variables for the first 90 min. All data were synchronized at the start of induction of anesthesia. Figure 2 shows the mean values of all the investigated variables with a time interval of 1 min.

MAP decreased significantly from 109 (16) to 83 (14) mmHg (*p* <0.05) after induction and remained stable afterwards. No inadvertent severe hypertension following prophylactic norepinephrine administration was observed at induction (Figure 1A). Heart rate also decreased upon induction of anesthesia from 73 (12) to 54 (8) bpm (*p* <0.05) but remained stable afterwards (56 (9) bpm at 60 min; non-significant vs. 20 min). After an initial drop in cardiac index from 3.0 (0.7) L min^-1^ m^-2^ at induction to 2.1 (0.4) L min^-1^ m^-2^ at 20 min thereafter (*p* <0.05), it gradually increased again to 2.4 (0.4) L min m^-2^ (*p* <0.05) at 60 min. StO_2_ increased significantly within minutes after induction of anesthesia from 83 (6)% before induction to 86 (4)% at 20 min after induction of anesthesia (*p* <0.05) to remain stable thereafter (87 (4)% at 60 min after induction of anesthesia). The mean (SD) BIS value dropped from 95 (4) before induction to 44 (9) after 20 min (*p* <0.05) and remained stable afterwards (mean BIS 44 (10) at 60 min).

**Figure 3:**
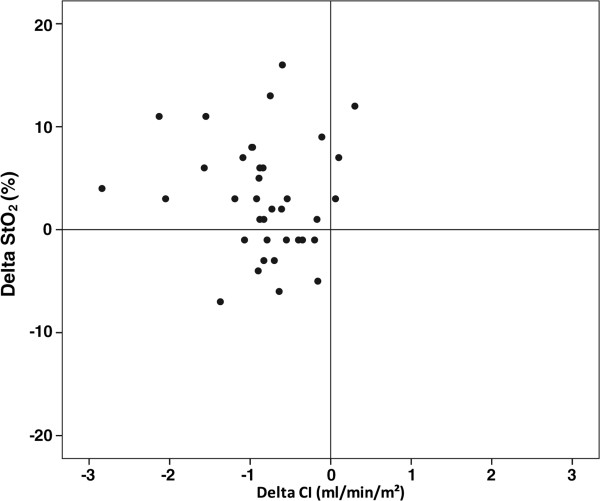
**The change in CI and StO**_**2 **_**between pre-anesthesia level and 20 min after induction.**

Figure 3 shows the changes in CI and StO_2_ from pre-anesthesia level to 20 min after induction in a scatterplot. Accordingly, a majority of patients have an increased StO_2_ despite decreased CI. There was no significant correlation between CI and StO_2_ values.

### Quality of surgical conditions

Complete akinesia was realized in all patients in the period between induction of anesthesia and the end of surgery, while no additional relaxant was administered after intubation and 90% of the cases lasted longer than 45 min. No neuromuscular reversal agents were needed.

The median (range) extubation time was 311 (72–600) s with an interquartile range of 253–386 s. None of the patients exhibited a coughing reflex upon extubation and none of the patients reported awareness or unpleasant experiences in the peri-anesthetic period upon explicit questioning 2 h after extubation.

## Discussion

In this prospective study in 40 patients, we have shown that during a high-antinociceptive balanced general anesthesia with propofol and remifentanil, goal-directed fluid administration and vasopressor support with norepinephrine results in a hemodynamically stable procedure with preservation of tissue oxygen saturation. This study performed in patients undergoing surgery with minimal noxious stimuli most of the time allows optimal quantification of the diverse effects of the anesthetic management with minimal interference of the surgical procedure. The combination of propofol/remifentanil, reported to provide an optimal depth of anesthesia, was also selected to produce a level of analgesia providing tolerance to laryngoscopy in >80% of cases (ED80) [2]. However, administration of analgesics to reach this high probability of tolerance to laryngoscopy requires a corresponding decrease in hypnotics in order to prevent excessive depth of anesthesia. Low BIS values (less than 30–40) are indicative of burst suppression [22], which is not considered a physiological EEG pattern, and has been correlated with poor outcome [16,23]. An appropriate decrease in propofol level is adequately reflected in a fitting mean (SD) BIS value of 44. This high level of antinociception proved to provide excellent analgesic and hypnotic conditions during surgery while also preventing intra- or postoperative events that might lead to increases in intra-ocular pressure or pharyngeal reflexes albeit allowing quick extubation with eventless recovery.

### Critique of the methods

Firstly, the current study was neither designed as a randomized controlled trial nor has there been a control group. The study was nonetheless aimed to elucidate the effects of a balanced high-antinociceptive general anesthesia on tissue oxygen saturation in a clinical setting, for which an observational study would be the most appropriate.

Secondly, the current study was performed solely in patients undergoing ophthalmic surgery and therefore we cannot draw firm conclusions on the question whether this anesthetic regimen would be applicable during other types of surgery. Nevertheless, we speculate that in other surgical procedures associated with intermittent painful stimuli but requiring absolute akinesia (e.g., rigid laryngoscopy) and subsequently requiring rapid recovery with a fully responsive and cooperative patient, the investigated high-antinociceptive balanced anesthesia might be of particular use. Further studies should address this issue.

While invasive measurements are conventionally preferred to continuously assess hemodynamic variables, we used the non-invasive Nexfin® device, for which accuracy and precision of MAP measurements were recently shown, to meet the Association for the Advancement of Medical Instrumentation criteria in patients undergoing thoracic surgery [24] and also, were shown to be not inferior to noninvasive cuff manometric measurement of MAP in hemodynamically stable patients under general anesthesia [10]. This device also measures CO with a percentage error (PE) between 23% and 26% compared to thermodilution derived CO measurements as reported for patients who are awake before and after coronary artery bypass surgery [25]. Another study [14], investigating the influence of phenylephrine infusion on Nexfin® CO measurements, found a high concordance (94%) between phenylephrine-induced changes in CO measured by Nexfin® and by esophageal Doppler. The PE found in this study was however relatively high with a PE of 33% before and 42.5% after phenylephrine infusion, suggesting a reduced accuracy after vasopressor administration. Yet, Nexfin® CO accuracy still requires further investigation in patients under general anesthesia and in situations such as hemodynamic instability or during infusion of (other) vasoactive drugs, e.g., norepinephrine. Nevertheless, this device allows continuous and non-invasive monitoring of blood pressure and flow without adding additional risk to the patient. Additionally, recent technologic advances have also allowed the ability to calculate dynamic preload variables, such as pulse pressure and stroke volume variation, in an automated continuous noninvasive fashion [18], yet with a reported [26] higher accuracy in assessing fluid responsiveness than clinical ∆POP assessment, as was performed in the current study.

Finally, the site and technology to determine tissue oxygenation may have some limitations. In particular, myoglobin can have relatively high oxygen saturation even in case of tissue hypoperfusion and decreased oxygenation, and it could be a major contributor to the readings obtained. As a consequence, there is a debate on which site should be preferred to accurately measure StO_2_[27]. However, the systematic increase in StO_2_ relative to baseline we observed in the current study convincingly demonstrates a favorable mixed effect of the anesthesia on global StO_2_.

### Quality of surgical conditions and emergence from anesthesia

In many surgical situations, a balanced high-antinociceptive general anesthesia is indicated, and a combination of propofol/remifentanil is appreciated. Based on the pharmacodynamic principle of equivalent anesthetic depth at different ratios of hypnotic/opiate, the chosen combination of propofol/remifentanil induced a sufficient level of anesthesia—reflected in normal BIS values between 40–60—although with a relatively higher contribution of remifentanil [2], resulting in a higher tolerance for sudden noxious stimuli. The favorable pharmacokinetic profile of this remifentanil/propofol combination resulted in a short median time to extubation—defined as the time interval between stopping the syringe pumps and the moment of extubation—of 311 s with low variability, together with optimal recurrence conditions: the fast elimination of propofol with still adequate analgesia at extubation results at the moment of endotracheal extubation in a complete suppression of potentially deleterious coughing reflexes and appropriate cooperation in all patients.

### Hemodynamics and tissue oxygenation

Anesthesia was induced with propofol, 1–3 mg kg^-1^, and remifentanil, 1 μg kg^-1^: a combination that can acutely decrease vascular tonus, heart rate, and cardiac output with resulting *relative* hypotension [28], which was also observed in the current study (Figure 2).

Firstly, in order to preserve cardiac preload, 13/40 patients were administered fluid as the ∆POP value was >10%, indicating fluid responsiveness, i.e., cardiac preload dependency.

Secondly, the decrease in CI following anesthesia induction is largely attributable to the (remifentanil-induced) decrease in heart rate (Figure 2), while stroke volume itself was relatively maintained probably because of both increased cardiac filling time and anesthesia-induced peripheral vasodilation and reduced cardiac afterload. The decrease in blood pressure after induction of anesthesia was anticipated with a single bolus of 10 μg norepinephrine at induction and a concomitantly started background infusion of norepinephrine. Most importantly, the administration of norepinephrine at the reported dose, in combination with goal-directed fluid optimization, was able to preserve a MAP at 80% of individual baseline values with a benign decrease in CO and without exerting adverse effects on StO_2_.

A major concern of combining potent hypnotics/analgetics with a vasopressor is a negative end-effect on cardiac output and ultimately on tissue oxygenation. Therefore, a rational dosing is essential to obtain the desired intubation conditions and surgical stability, with optimal preservation of hemodynamic homeostasis. The stable BIS value within the recommended range reflects an adequate and reliable depth of hypnosis and demonstrates that a strong antinociceptive effect can be safely obtained if combined with an adequately adapted administration of propofol. Secondary, while the slight increase in tissue oxygenation is reassuring, a significant decrease in CI between awake and post-induction state demonstrates the potent hemodynamic effects of induction of even a balanced anesthesia. This decrease in CI is mainly based on a decrease in systemic resistance and heart rate and a preserved stroke volume. The resulting decrease in cardiac oxygen consumption, combined with a preserved peripheral tissue oxygenation, suggests that a CI decrease to this extent can be considered acceptable for induction of anesthesia. The subsequent increase in CI and stable MAP and StO_2_ demonstrates that the moderate continuous doses of vasopressor are sufficient and appropriate to sustain hemodynamic stability during guaranteed optimal surgical conditions. However, the potent effects of the vasopressors must be reckoned with and must be considered in respect to other cardiovascular comorbidities. In addition, a more subtle administration of hypnotics/analgetics, such as based on TCI models, may be more appropriate in vulnerable patients.

While this desired MAP may appear relatively high for some clinicians, several reports [15,16] acknowledge the utmost importance of a preserved MAP up this value to prevent adverse outcome. Importantly, care must be taken that potent vasopressors like norepinephrine are administered with respect to appropriate safety measures, such as the preferable use of a dedicated line and avoidance of inadvertent boluses.

For patients under general anesthesia, StO_2_ values around 80% are considered within the normal range [29]. The increase in StO_2_ to 86(4)% in our study was most probably caused by peripheral vasodilation due to the combination of propofol and remifentanil. This finding is in contrast to a previous study in which no differences in StO_2_ were found before and after induction of anesthesia [29]. The use of sufentanil in this previous study may account for this difference. Nevertheless, StO_2_ is believed to be a flow-dependent variable [9]. Furthermore, it has been shown to be independent of MAP and remained almost constant at different levels of MAP [30]. It has however not yet been investigated whether thenar StO_2_ values also correlate with oxygen saturation of tissues of vital importance (e.g., heart, brain, kidney, liver). Subsequently, although the data show favorable values of thenar StO_2_, indicating adequate peripheral tissue oxygenation (at least in the range of MAP and CI values observed), ‘macro’ hemodynamic values such as CI, heart rate, and MAP are of great importance for assuring patient safety.

## Conclusions

A high-antinociceptive balanced anesthesia with goal-directed fluid and norepinephrine administration preserved tissue oxygen saturation while other hemodynamic variables remained within a clinically acceptable range. Furthermore, this approach provided reliable surgical conditions, complete akinesia, and swift postoperative recovery.

## Competing interests

The authors declare that they have no competing interests.

## Authors’ contributions

JJV was involved in the patient inclusion, data recording, data analysis and interpretation, and manuscript writing. MP participated in the data analysis and manuscript writing. LNH participated in the data analysis, pharmacologic modeling, data interpretation, and manuscript writing. VWRDL was involved in the patient inclusion and manuscript writing. MMRFS and TWLS participated in the data interpretation and manuscript writing. AFK was involved in the patient inclusion and patient care, data recording, data analysis and interpretation, and manuscript writing. All authors read and approved the final manuscript.
